# CREATING STRENGTH IN AGE: THE ROLE OF ARTS, ETHICS, AND HUMANITIES IN GERONTOLOGY EDUCATION

**DOI:** 10.1093/geroni/igz038.111

**Published:** 2019-11-08

**Authors:** Pamela Saunders

**Affiliations:** Georgetown University, Washington, District of Columbia, United States

## Abstract

What are the necessary and ideal components of an interdisciplinary master’s degree program in gerontology? What is the role of arts, humanities, and ethics to teach students and future leaders the power of social networks? This paper presents a case study of one academic program. Data are semi-structured interviews with students and faculty analyzed using content analysis to discover themes. In 2018, inspired by aging demographics, Georgetown University launched a one-year, interdisciplinary MS degree in Aging & Health. Informed by AGHE competencies including arts, ethics, and humanities, the program provides theoretically and scientifically grounded content, hands-on experience, and professional engagement. One core course specifically focuses on arts, ethics, and humanities, and provides a basis for students to see interdisciplinary links across the curriculum. Findings includes themes that illustrate the value of harnessing the power of arts, humanities, and ethics, including a diverse, and intergenerational cohort, and students’ cultural value systems.

